# IL-6 receptor blockade does not slow β cell loss in new-onset type 1 diabetes

**DOI:** 10.1172/jci.insight.150074

**Published:** 2021-11-08

**Authors:** Carla J. Greenbaum, Elisavet Serti, Katharina Lambert, Lia J. Weiner, Sai Kanaparthi, Sandra Lord, Stephen E. Gitelman, Darrell M. Wilson, Jason L. Gaglia, Kurt J. Griffin, William E. Russell, Philip Raskin, Antoinette Moran, Steven M. Willi, Eva Tsalikian, Linda A. DiMeglio, Kevan C. Herold, Wayne V. Moore, Robin Goland, Mark Harris, Maria E. Craig, Desmond A. Schatz, David A. Baidal, Henry Rodriguez, Kristina M. Utzschneider, Hendrik J. Nel, Carol L. Soppe, Karen D. Boyle, Karen Cerosaletti, Lynette Keyes-Elstein, S. Alice Long, Ranjeny Thomas, James G. McNamara, Jane H. Buckner, Srinath Sanda, for the ITN058AI EXTEND Study Team

**Affiliations:** 1 Center for Interventional Immunology and Diabetes Program, Benaroya Research Institute, Seattle, Washington, USA.; 2 Immune Tolerance Network, Seattle, Washington, USA.; 3 Rho, Inc, Durham, North Carolina, USA.; 4 University of California, San Francisco, San Francisco, California, USA.; 5 Stanford University, Stanford, California, USA.; 6 Joslin Diabetes Center, Harvard Medical School, Boston, Massachusetts, USA.; 7 Sanford Health, San Jose, California, USA.; 8 Vanderbilt University, Nashville, Tennessee, USA.; 9 University of Texas, Southwestern, Dallas, Texas, USA.; 10 University of Minnesota, Minneapolis, Minnesota, USA.; 11 Children’s Hospital of Philadelphia, University of Pennsylvania, Philadelphia, Pennsylvania, USA.; 12 University of Iowa, Iowa City, Iowa, USA.; 13 Riley Children’s Hospital, Indiana University, Indianapolis, Indiana, USA.; 14 Yale University, New Haven Connecticut, USA.; 15 University of Missouri, Kansas City, Kansas City, Missouri, USA.; 16 Columbia University, New York, New York, USA.; 17 Children’s Health Queensland Hospital, South Brisbane, Australia.; 18 University of Sydney, Sydney New South Wales, Australia.; 19 University of Florida, Gainesville, Florida, USA.; 20 University of Miami, Coral Gables, Florida, USA.; 21 University of South Florida, Tampa, Florida, USA.; 22 VA Puget Sound and the University of Washington, Seattle, Washington, USA.; 23 University of Queensland, Queensland, Brisbane, Australia.; 24 National Institute of Allergy and Infectious Diseases, NIH, Bethesda, Maryland, USA.; 25 The ITN058AI EXTEND Study Team members are detailed in the Supplemental Acknowledgments.

**Keywords:** Endocrinology, Immunology, Beta cells, Diabetes, T cells

## Abstract

**Background:**

IL-6 receptor (IL-6R) signaling drives development of T cell populations important to type 1 diabetes pathogenesis. We evaluated whether blockade of IL-6R with monoclonal antibody tocilizumab would slow loss of residual β cell function in newly diagnosed type 1 diabetes patients.

**Methods:**

We conducted a multicenter, randomized, placebo-controlled, double-blind trial with tocilizumab in new-onset type 1 diabetes. Participants were screened within 100 days of diagnosis. Eligible participants were randomized 2:1 to receive 7 monthly doses of tocilizumab or placebo. The primary outcome was the change from screening in the mean AUC of C-peptide collected during the first 2 hours of a mixed meal tolerance test at week 52 in pediatric participants (ages 6–17 years).

**Results:**

There was no statistical difference in the primary outcome between tocilizumab and placebo. Immunophenotyping showed reductions in downstream signaling of the IL-6R in T cells but no changes in CD4 memory subsets, Th17 cells, Tregs, or CD4^+^ T effector cell resistance to Treg suppression. A DC subset decreased during therapy but regressed to baseline once therapy stopped. Tocilizumab was well tolerated.

**Conclusion:**

Tocilizumab reduced T cell IL-6R signaling but did not modulate CD4^+^ T cell phenotypes or slow loss of residual β cell function in newly diagnosed individuals with type 1 diabetes.

**Trial Registration:**

ClinicalTrials.gov NCT02293837.

**Funding:**

NIH National Institute of Diabetes and Digestive and Kidney Diseases (NIDDK) and National Institute of Allergy and Infectious Diseases (NIAID) UM1AI109565, UL1TR000004 from NIH/National Center for Research Resources (NCRR) Clinical and Translational Science Award (CTSA), NIH/NIDDK P30DK036836, NIH/NIDDK U01DK103266, NIH/NIDDK U01DK103266, 1UL1TR000064 from NIH/NCRR CTSA, NIH/National Center for Advancing Translational Sciences (NCATS) UL1TR001878, UL1TR002537 from NIH/CTSA; National Health and Medical Research Council Practitioner Fellowship (APP1136735), NIH/NIDDK U01-DK085476, NIH/CTSA UL1-TR002494, Indiana Clinical and Translational Science Institute Award UL1TR002529, Vanderbilt Institute for Clinical and Translational Research UL1TR000445. NIH/NCATS UL1TR003142, NIH/CTSA program UL1-TR002494, Veteran Affairs Administration, and 1R01AI132774.

## Introduction

There is a significant unmet need for disease-modifying therapy in type 1 diabetes (T1D). Despite the advent of modified insulins and new insulin delivery technologies, disease management remains suboptimal, and patients continue to experience disease-associated morbidity, day-to-day emotional and financial burdens, and reduced life expectancy (1–3). The goal of disease-modifying therapy in T1D, similar to other autoimmune diseases, is to modulate the autoimmune process instead of treating symptoms (i.e., hyperglycemia).

Over the past 2 decades, 7 trials of immune-modulating therapies have shown efficacy in preserving insulin secretion after clinical diagnosis. These studies targeted adaptive immune cells (such as anti–B cell therapy with rituximab and anti–T cell therapy with teplizumab, antithymocyte globulin, and alefacept), blocked costimulation pathways (abatacept), and inhibited cytokines (anti–IL-21 and anti-TNF; refs. 4–10).

IL-6 is a pleiotropic cytokine involved in both innate and adaptive immune responses. IL-6 belongs to a family of cytokines sharing a common receptor subunit, gp130 (11). Classical IL-6 signaling occurs after IL-6 binds to membrane-bound IL-6 receptor (IL-6R) and associates with gp130, forming an active ligand-receptor complex and activating, via phosphorylation, the JAK/signal transducer and activator of transcription protein 3 (JAK/STAT3) cascade (12). Targeting IL-6 in T1D would be rational based on the immunology of the disease. Both T helper 17 (Th17) cells and T regulatory cells (Tregs) contribute to the development of T1D, with IL-6 augmenting development of pathogenic Th17 effector cells and blocking the development and function of suppressive Tregs (13–15). In addition, T effector cells from T1D individuals are hyperresponsive to IL-6 and resistant to suppression by Tregs in vitro (16). Also, signaling through the IL-6R appears to play a role in risk of developing T1D based on the observation that a functional *IL-6R* variant impairs classical IL-6 signaling and may protect against the development of T1D (17).

Tocilizumab is a monoclonal antibody that blocks the IL-6R. It has been shown to be effective in patients with juvenile idiopathic arthritis and rheumatoid arthritis and is currently approved for use in children as young as 2 years of age for the treatment of polyarticular juvenile idiopathic arthritis, systemic juvenile idiopathic arthritis, and cytokine release syndrome (18, 19). Given the mechanistic rationale for the role of IL-6 signaling in T1D and that other effective rheumatoid arthritis drugs (abatacept and TNF-α blockers) have shown benefit in T1D, we hypothesized that blockade of the IL-6 pathway with tocilizumab would lead to clinical improvements in T1D (4, 10). We conducted a multicenter, randomized, placebo-controlled trial in individuals with newly diagnosed T1D. Our primary outcome was a change in residual insulin secretion at 1 year as measured by 2-hour C-peptide mean area under the curve (mAUC) in pediatric participants following 6 months of therapy. While children ages 6 to 17 years were the primary efficacy population in this study, a cohort of adults was enrolled initially for a safety evaluation before opening the study to the pediatric group. Additional aims of the study were to evaluate the safety of tocilizumab treatment in T1D and to examine changes in immune cell subsets after tocilizumab therapy.

## Results

### Participants and disposition.

**Figure 1:**
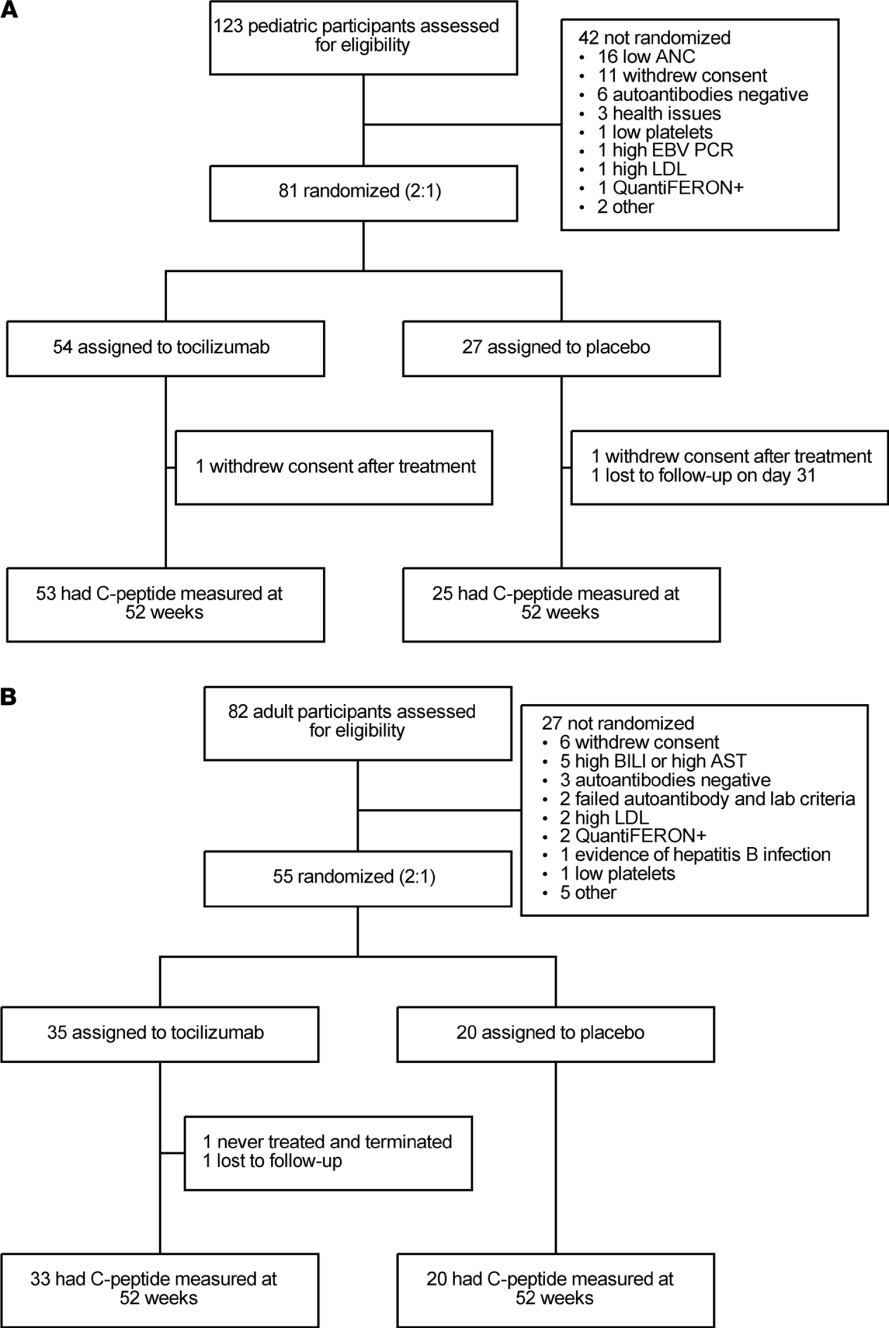
CONSORT diagrams for both cohorts. (**A**) Pediatric participants and (**B**) adult participants. ANC, absolute neutrophil count; BILI, bilirubin; AST, aspartate aminotransferase.

Adults and pediatric patients screened and randomized are shown (Figure 1). A total of 6 tocilizumab-treated participants (3 adult and 3 pediatric) discontinued therapy early, and a total of 2 placebo-treated patients discontinued prior to completing the course of study medication (1 adult and 1 pediatric). The pediatric and adult cohorts had, respectively, 54 and 34 modified intention to treat (mITT) participants in the tocilizumab arm and 27 and 20 mITT participants in the placebo arm.

In both the adult and pediatric cohorts, most participants were White (Table 1). There were more men than women in the adult cohort. On average, participants in both cohorts had excellent glucose control at study entry as evidenced by the mean HbA1c values of less than 7%.

### Safety profile of tocilizumab in T1D.

A total of 3 treatment-emergent serious adverse events (SAEs) occurred in the tocilizumab groups and 4 in the placebo groups (including both pediatric and adult cohorts). None of the SAEs in the tocilizumab group were considered related to study therapy. There was no difference in the overall percentage of treatment-emergent adverse events (AEs) between groups in both age cohorts (Table 2). Infection rates were comparable between treatment groups. As expected, the rate of infusion reactions in both cohorts was higher in the tocilizumab compared with the placebo groups (pediatric: *P* = 0.296, adult: *P* = 0.145, pooled: *P* = 0.027) (Table 2). There were no differences over time in cholesterol, HDL, or LDL between treatment groups in either the adult or pediatric cohort (Supplemental Figure 1; supplemental material available online with this article; https://doi.org/10.1172/jci.insight.150074DS1).

### Tocilizumab does not affect residual β cell function, insulin usage, or glucose control.

**Figure 2:**
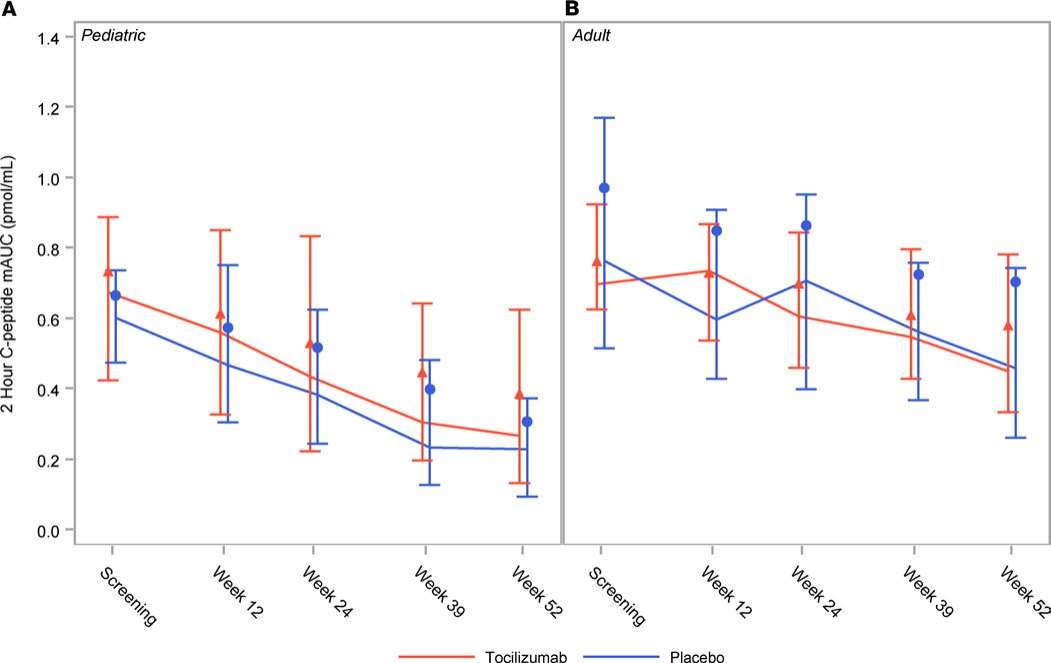
Tocilizumab does not affect 2-hour C-peptide mAUC. Markers represent the means, lines connect the medians, and error bars represent the 25th and 75th percentiles of C-peptide mAUC collected during the first 2 hours of the mixed meal tolerance test (MMTT) shown over the first year for (**A**) the pediatric cohort and (**B**) the adult cohort. ANCOVA models and mixed model analysis did not detect any statistically significant differences between the treatment groups at key time points.

There was no difference in the 2-hour C-peptide mAUC at week 52 between the tocilizumab- and placebo-treated groups in either the pediatric or adult cohort (Figure 2). The least squares means from the primary endpoint model (pediatric participants) for the change from screening to week 52 in 2-hour C-peptide mAUC were –0.337 (95% CI: –0.39, –0.28) for the tocilizumab arm and –0.391 (95% CI: –0.47, –0.31) for the placebo arm (*P* = 0.277). Mixed model analysis of 2-hour C-peptide mAUC did not find any difference between the treatment groups at screening or over time for each age cohort. Participants over 12 years of age completed a 4-hour MMTT. No difference in 4-hour MMTT mAUC between baseline and week 52 was observed between treatment arms in both the pediatric and adult participants (Supplemental Figure 2). Patients were genotyped for *IL6R* SNPs (rs4129267 and rs61812598) in 100% linkage disequilibrium with the rs2228145 variant that may confer protection against T1D (17) to determine if genotype affected the primary outcome. Genotype proportions for the adult and pediatric patients for each variant were not abnormally distributed (Supplemental Table 2). No relationship between genotypes and rate of decline in insulin secretion was observed (data not shown).

**Figure 3:**
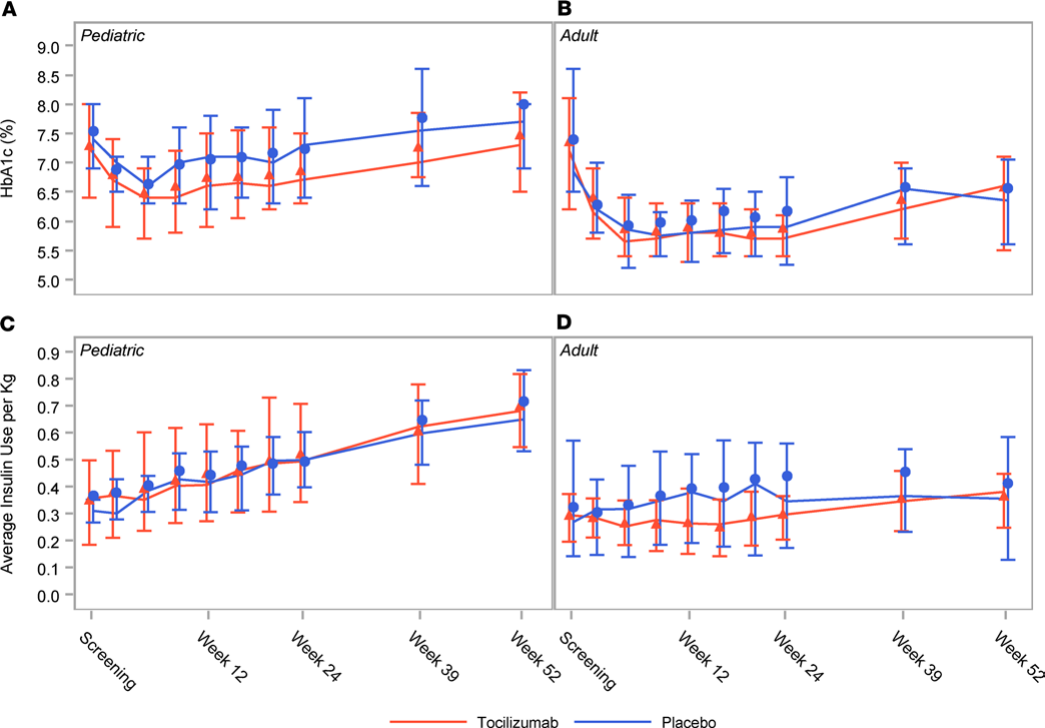
Tocilizumab does not affect insulin usage and glucose control. Markers represent the means, lines connect the medians, and error bars represent the 25th and 75th percentiles. HbA1c values for (**A**) the pediatric cohort and (**B**) the adult cohort. Average daily insulin usage expressed as total daily units/kg for (**C**) the pediatric cohort and (**D**) the adult cohort. ANCOVA models and mixed model analysis did not detect any statistically significant differences between the treatment groups at key time points.

No significant differences were seen between treatment arms with respect to average total daily insulin usage or HbA1c in either the pediatric or adult cohort (Figure 3). The proportion of participants with at least 1 major hypoglycemic event was not different between the treatment groups for pediatric participants (*P* = 0.634), adults (*P* = 0.329), or the cohorts combined (*P* = 0.847).

Insulin sensitivity was also assessed in a subset of participants who completed the frequently sampled intravenous glucose tolerance testing (FSIVGTT). Since IL-6 may regulate peripheral insulin resistance, it was hypothesized that blockade of the IL-6 pathway with tocilizumab would result in improved insulin sensitivity. A total of 19 participants elected to participate, and 11 participants had adequate data for modeling at the 3 visits. No change in insulin sensitivity was noted in any active drug participant (Supplemental Figure 3).

In addition, continuous glucose monitoring (CGM) data were collected when available. Longitudinal CGM data for pediatric participants are shown (Supplemental Table 1). There was no clear relationship between C-peptide mAUC and CGM data throughout the study.

### Anti–IL-6R transiently reduces signaling in T cell subsets but does not alter T cell phenotypes.

**Figure 4:**
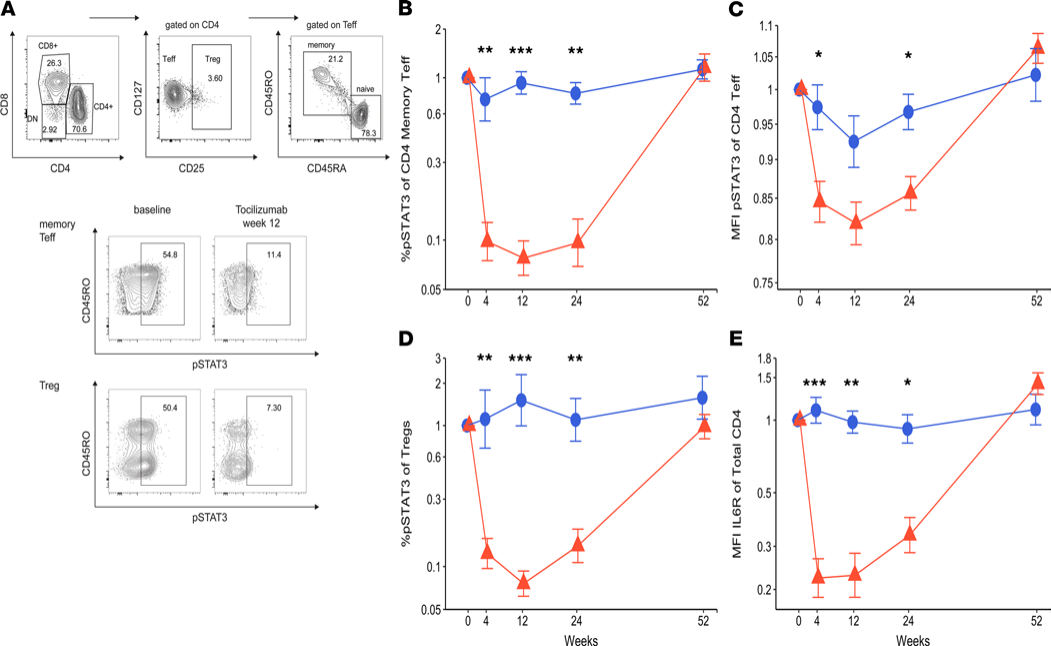
Tocilizumab impairs IL-6R signaling in T cells. (**A**) Gating strategy of CD4^+^ Tregs and memory Teff cells and phosphorylated STAT3 (p-STAT3) expression after in vitro IL-6 stimulation, at baseline and week 12 of treatment of a representative treated patient. Longitudinal fold changes from baseline for (**B**) percentage of CD4^+^ Tem cells expressing p-STAT3, (**C**) MFI of p-STAT3 in CD4^+^ Tem cells, (**D**) percentage of Tregs expressing p-STAT3, (**E**) MFI of IL-6R in total CD4. *Y* axis scales are log transformed. Mean fold changes from baseline are presented at each visit. Error bars display SEM. *P* values were calculated using repeated measure 2-way ANOVA model. Statistically significant comparisons are shown with asterisks (***P < 0.0001; **P ≤ 0.001; *P ≤ 0.05). DN, double negative.

We analyzed longitudinal blood samples from trial participants to understand the immunological effects of tocilizumab given its lack of clinical efficacy in this study. We first determined whether tocilizumab suppressed signaling in T cells downstream of the IL-6R. Activation of the IL-6R on T cells results in phosphorylation of the transcription factor STAT3 (12). In our trial, monthly tocilizumab dosing over the 24-week treatment period led to significant and rapid reductions of phosphorylated STAT3 in memory CD4^+^ T effector (Teff) cells (CD4^+^CD45RO^+^CD45RA^–^, Figure 4, A–C) compared with placebo. However, 6 months after stopping the drug, at week 52, STAT3 phosphorylation in the tocilizumab group returned to levels comparable to those of the placebo group. Tocilizumab therapy also reduced STAT3 phosphorylation in CD4^+^ Tregs (CD4^+^CD25^hi^; Figure 4D). In addition, we observed that surface IL-6R expression on CD4^+^ T cells decreased during therapy with tocilizumab, consistent with the known mechanism of the drug (Figure 4E). These experiments confirmed that tocilizumab blocked IL-6R signaling in T cells.

**Figure 5:**
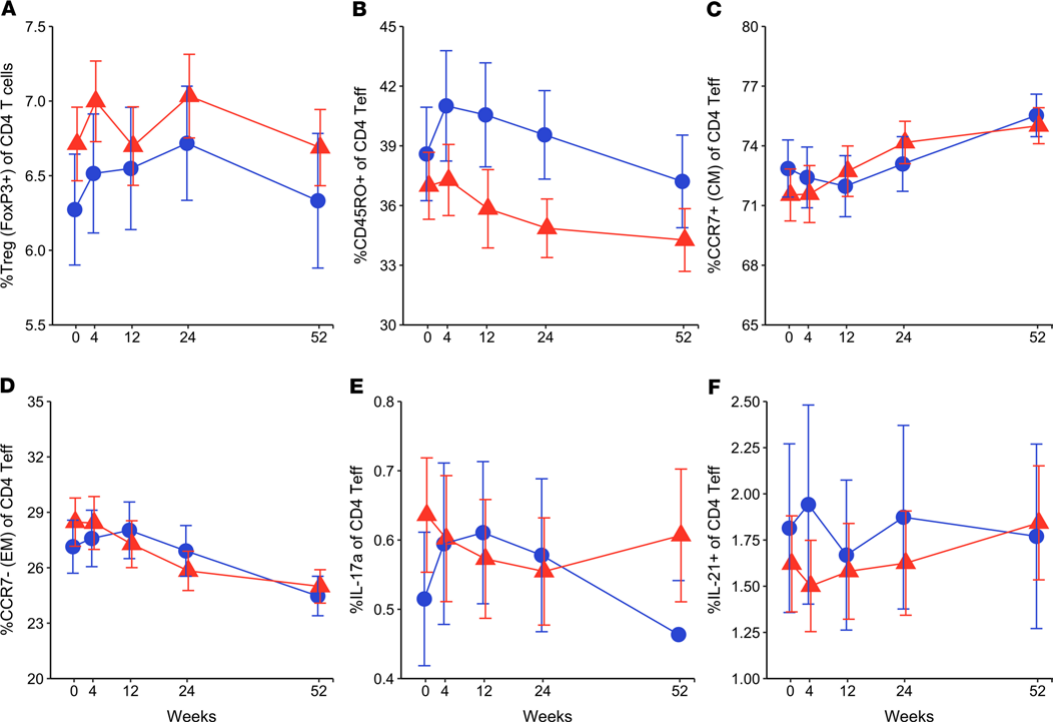
Tocilizumab does not alter frequency of CD4^+^ T cell subsets. (**A**) Percentage of Tregs in total CD4. Percentage of (**B**) memory, (**C**) central memory, (**D**) effector memory, (**E**) Th17 (IL-17a^+^), (**F**) Tfh (IL-21^+^) in CD4^+^ Teff cells. *Y* axis scales are log transformed. Mean values are presented at each visit. Error bars display SEM. Repeated measure 2-way ANOVA did not detect any statistically significant differences between the treatment groups at key time points.

**Figure 6:**
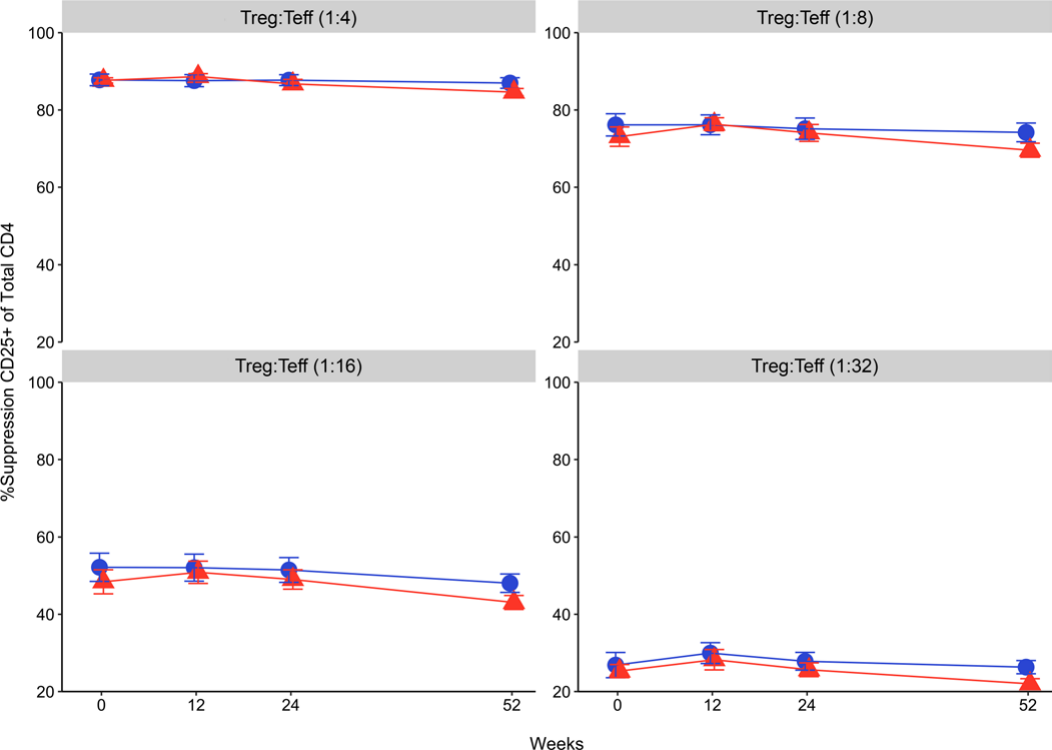
Tocilizumab does not alter Teff cell response to Treg suppression. Percentage suppression of CD25 expression on Teff cells by Tregs. For all time points, Teff cells were cocultured with EF670-labeled Tregs and anti-CD3/anti-CD28 Dynabeads (1:28 beads/Teff) for 48 hours. Four Treg/Teff ratios were tested: 1:4 (top left), 1:8 (top right), 1:16 (bottom left), and 1:32 (bottom right). For flow cytometry, Teff cells were in the EF670^–^ gate and stained with anti-CD25 PE-Cy7. Percentage suppression was calculated as follows: *s* = ([*a* – *b*]/*a*) × 100, where *a* is the percentage CD25^+^ in the absence of Tregs and *b* is the percentage of CD25^+^ in the presence of Tregs. Data are represented as mean ± SEM. There were no statistical differences between placebo and tocilizumab at any Treg/Teff ratio or any time point. Repeated measures 2-way ANOVA did not detect any statistically significant differences between the treatment groups at key time points.

Despite impaired IL-6 signaling, no changes were observed in the phenotype and function of circulating T cells. There was no effect of tocilizumab on the percentages of the CD4^+^ Treg (CD4^+^FoxP3^+^), total CD4 memory, or CD4 memory subsets (CD4^+^FoxP3^–^CD45RO^+^CCR7^+^ for central memory and CD4^+^FoxP3^–^CD45RO^+^CCR7^–^ for Tem cells; Figure 5, A–D). We also found no reductions in Th17 (CD4^+^FoxP3^–^CD45RO^+^IL-17a^+^) or T follicular helper (Tfh) (CD4^+^FoxP3^–^CD45RO^+^IL-21^+^) subsets (Figure 5, E and F). Although prior in vitro studies suggested that IL-6 impairs CD4^+^ Teff susceptibility to suppression by Tregs (20, 21), no differences in suppression were seen between the tocilizumab and placebo groups (Figure 6).

### Anti–IL-6R transiently reduces conventional type 2 DCs.

**Figure 7:**
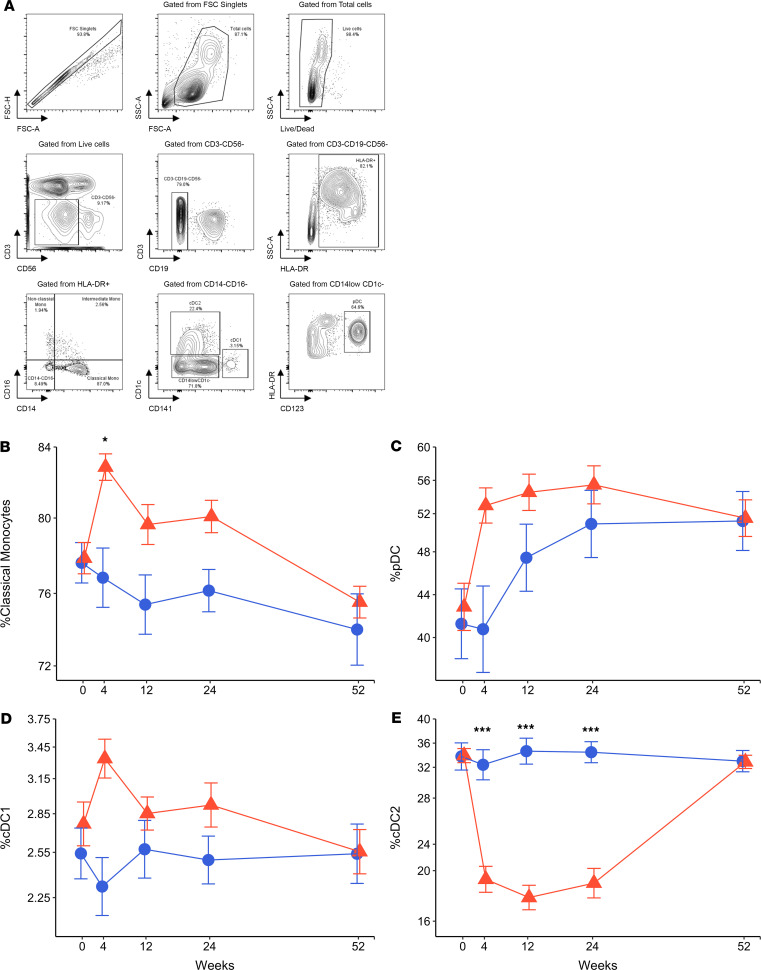
Tocilizumab changes frequencies of DC populations. (**A**) Gating strategy for DC subsets (plasmacytoid DCs, pDCs; conventional type 1 DCs, cDC1s; and conventional type 2 DCs, cDC2s) and classical, nonclassical, and intermediate monocytes. (**B**) Percentage of classical monocytes in HLA-DR^+^ cells. (**C**) Percentage of pDCs (CD123^+^HLA-DR^+^) in CD14^lo^CD1c^–^ cells. (**D**) Percentage of cDC1s (CD141^hi^CD1c^–^) and (**E**) cDC2s (CD141^lo^CD1c^+^) in CD14^–^CD16^–^cells. *Y* axis scales are log transformed and presented in actual scale. Mean values are presented at each visit. Error bars display SEM. *P* values were calculated using repeated measure 2-way ANOVA model. Statistically significant comparisons are shown with asterisks (***P < 0.0001; *P ≤ 0.05).

Given the importance of IL-6 in myeloid cell function, we studied whether tocilizumab affected monocytes and DCs. Classical monocytes (CD3^–^CD19^–^CD56^–^HLA-DR^+^CD14^hi^CD16^–^) increased during the early phase of treatment with tocilizumab, but the increase did not persist over time (Figure 7, A and B). Tocilizumab recipients had transient but not statistically significant increases in the frequency of pDCs (CD3^–^CD19^–^CD56^–^HLA-DR^+^CD14^–^CD16^–^CD141^lo^CD1c^–^CD123^+^) and cDC1s (CD3^–^CD19^–^CD56^–^HLA-DR^+^CD14^–^CD16^–^CD141^hi^CD1c^–^; Figure 7, C and D). Unexpectedly, the percentage of cDC2s (CD3^–^CD19^–^CD56^–^HLA-DR^+^CD14^–^CD16^–^CD141^lo^CD1c^+^) was markedly decreased in active drug recipients during the 24-week treatment phase and returned to baseline levels after study drug withdrawal (Figure 7E). The frequency of cDC2s in the placebo group remained stable over time.

### Increased IL-6 and soluble IL-6R in anti–IL-6–treated participants.

**Figure 8:**
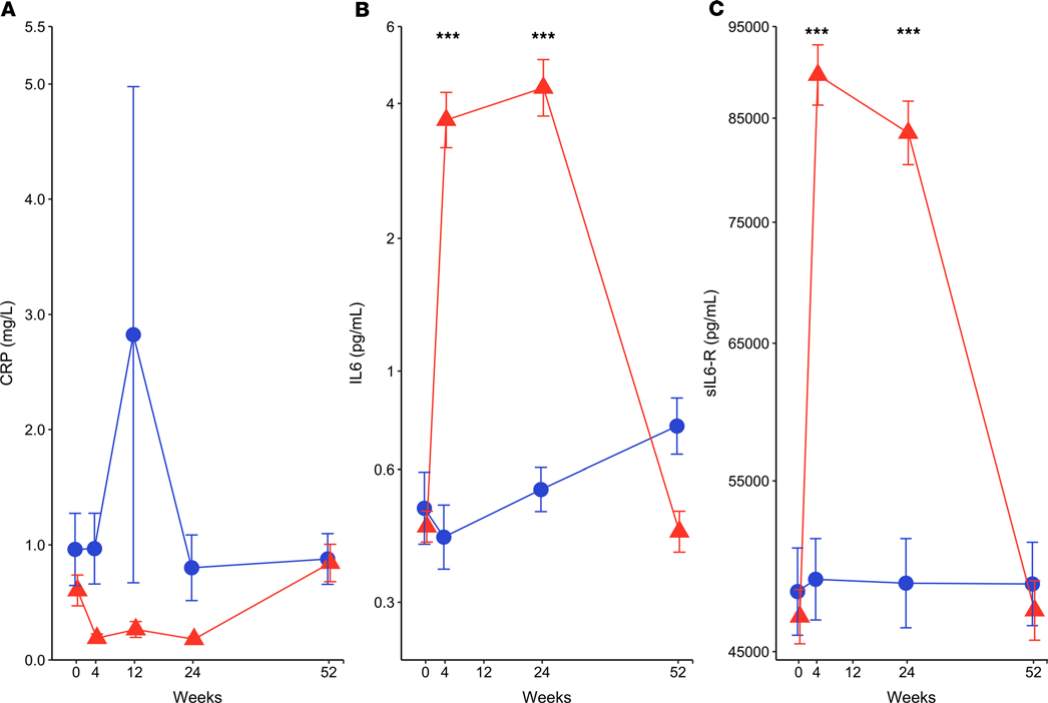
Increased serum IL-6 and IL-6R with tocilizumab therapy. (**A**) C-reactive protein, (**B**) IL-6, and (**C**) soluble IL-6R. *Y* axis scales are log transformed and presented in actual scale for IL-6 and IL-6R analytes. C-reactive protein is plotted in actual scale. Mean values are presented at each visit. Error bars display SEM. *P* values were calculated using repeated measure 2-way ANOVA model. (***P < 0.0001.)

We also analyzed serum markers of inflammation. C-reactive protein levels, although not significantly elevated in either group at baseline, decreased during therapy in the tocilizumab group compared with the placebo group (Figure 8A). Interestingly, we observed increases in IL-6 and soluble IL-6R (sIL-6R) in the serum of active drug recipients (Figure 8, B and C) that may have resulted from tocilizumab binding to sIL-6R. Other serum cytokines measured (IFN-α, TARC, MDC, MIP1α, MIP1β, IL-1b, IL-7, IL-10, IL-12, IL-15, IL-17A, IL-8, IFN-γ, TNF-α, IFN-β, and IL-27) did not show differences between the 2 treatment arms over time.

## Discussion

Results from this randomized, placebo-controlled trial demonstrated that IL-6R blockade with tocilizumab did not slow the loss of residual β cell function in children or adults with T1D in the first year after randomization. The high retention of participants in a well-powered clinical trial led to an unambiguous, but disappointing, clinical result.

Given the role of IL-6 in regulating T cell function and its importance in T1D, the results of this trial were surprising. Prior to this study, data from both animal models and humans supported the approach of IL-6 blockade in T1D. In murine models, IL-6 signaling reduces the frequency of FoxP3 Tregs in favor of expansion of Th17 cells, changes that reflect models of T1D pathogenesis (14, 22, 23). In addition, NOD mice treated with tocilizumab maintain euglycemia and have less demonstrable insulitis (24). Patients with T1D also seem to have evidence of increased IL-6 signaling. Myeloid cells from patients with T1D show increased secretion of IL-6 compared with control and type 2 diabetes patients (25). Phosphorylation of STAT3 is also increased in patients with T1D compared with control patients and results in resistance of Teff cells to Treg suppression (16, 21, 26). Additionally, a coding *IL-6R* variant, rs2228145, confers protection against the development of T1D in humans by promoting membrane shedding of IL-6R by the protease ADAM17 (a disintegrin and a metalloproteinase domain 17), suggesting that reduced IL-6R signaling may be protective (17, 27). Together these data strongly implicate the IL-6 pathway in T1D pathogenesis and predict that blockade of the IL-6R with tocilizumab would have some clinical benefit.

However, despite impairment of IL-6R signaling in this trial, expected changes in T cell phenotypes did not occur. In rheumatoid arthritis, tocilizumab therapy decreases Tfh frequency, and increases Treg frequency, but does not change the frequency of Th17 cells (28–30). The lack of effect on Th17 cells in the present study may have been predicted based on earlier in vitro work in human cells that showed that IL-1 receptor blockade, in combination with anti–IL-6 therapy, is needed to decrease Th17 cells (25). However, the EXTEND trial did not show changes in other T cell subsets that would have been predicted from trials in other autoimmune diseases. For diseases where tocilizumab has been effective (rheumatoid arthritis, polyarticular juvenile idiopathic arthritis, systemic juvenile idiopathic arthritis, and cytokine release syndrome), systemic inflammation is a feature of the disease and IL-6 levels are elevated. This is not the case in T1D and suggests that tocilizumab’s ability to modulate the T cells depends on an inflammatory milieu. In the context of other T1D trials using cytokine receptor antagonists that failed to show efficacy, such as the IL-1R antagonist anakinra, the broader approach of inhibiting inflammatory pathways with receptor blockers should be reevaluated (31).

Alternative signaling pathways may also help explain the lack of clinical efficacy. Classical IL-6 signaling involves IL-6 binding to membrane-bound IL-6R. However, cells may release sIL-6R either by the activity of the ADAM17 protease or secretion of an alternative splice product lacking a transmembrane domain (12). In a similar fashion, soluble gp130 (sgp130) is also present in the circulation and, together with sIL-6R, functions as a “cytokine sink,” limiting IL-6 activity. Excess amounts of IL-6 and sIL-6R may exceed levels of sgp130, resulting in activation of gp130-expressing cells via transactivation (32).

Despite the pharmacodynamic changes indicating marked IL-6R blockade (i.e., a nearly 10-fold reduction in phosphorylated STAT3 in T cells), the inherent redundancy of IL-6 signaling mechanisms may have prevented a complete blockade of the pathway with tocilizumab monotherapy. The parallel increases we observed in serum IL-6 and sIL-6R suggest that transactivation theoretically could have occurred in our trial. Tocilizumab treatment is known to saturate sIL-6R, prolonging the circulating half-life of sIL-6R and reducing consumption of IL-6, resulting in elevated serum IL-6 levels (33). In our study, there was close to a 10-fold increase in serum IL-6 at the end of tocilizumab therapy, but it is not clear if that degree of increased ligand availability would result in transactivation of the IL-6 pathway. It is also possible that global blockade of IL-6R may have directly affected the Treg pool, including a recently described Treg subset seen in patients with T1D that expresses high levels of IL-6R and exhibits suppressive function (34). While alternative signaling was possible in our trial, it does not reconcile the efficacy of tocilizumab in other autoimmune diseases, where similar changes in serum IL-6 and sIL-6R have been reported (33). It may be that complete systemic blockade with a receptor antagonist is not possible, and downstream blockade of the IL-6R/gp130 complex may be needed in T1D. Additionally, while combining tocilizumab with another anticytokine agent is appealing, such a strategy would require additional mechanistic rationale that incorporates the cause for tocilizumab’s lack of efficacy in T1D.

Besides the lack of modulation of the T cell pool, our trial also found reductions in cDC2s with tocilizumab treatment. Although reductions in DCs have been reported previously in tocilizumab-treated patients with rheumatoid arthritis (35), no previous tocilizumab trial to our knowledge has analyzed cDC subsets in detail or reported reductions in cDC2s. Two subsets of cDCs in humans exist, cDC1s and cDC2s, and their function seems context dependent. In the cancer microenvironment, cDC2s can recruit naive T cells to mount a Th1 response against malignant cells (36). In autoimmunity, cDC1s are involved in cross-presentation of antigen to CD8^+^ T cells while cDC2s present antigen to CD4 and may be more tolerogenic by promoting expansion of Tregs (37). It is possible that the failure in our study to increase Tregs, as seen in other tocilizumab trials, may have resulted from unexpected reductions in cDC2s. What role IL-6 plays in the homeostasis of cDC2s and whether this role is unique to patients with T1D remains to be clarified.

Finally, it is important to consider the nonimmunological functions of IL-6 in the context of understanding the trial outcomes. Paradoxical data exist about the role of IL-6 in metabolism. Animal data suggest that IL-6 mediates hepatic insulin resistance via SOCS3, which blocks autophosphorylation of the insulin receptor (38). In muscle, however, IL-6 enhances glucose uptake via glucose transporter 4 and increases fatty acid oxidation by upregulating AMPK (39). Additionally, it should be noted that IL-6 itself may have a direct role in impairing reactive oxygen species generated in β cells (40). In our study, no changes in insulin sensitivity were noted during the FSIVGTT, suggesting that the metabolic effects of IL-6 do not play a significant role in β cell function in T1D.

In conclusion, the use of tocilizumab did not improve clinical outcomes in patients with T1D. Tocilizumab’s use was associated with increases in sIL-6R and serum IL-6 and a reduction in cDC2s but did not alter Teff resistance or the frequencies of Th17, Tfh, and Treg subsets. The lack of response to tocilizumab in this study suggests that the role of IL-6 in T1D is complex. Therapeutic interventions targeting IL-6 in the future may be most beneficial in combination with therapies that synergize with the IL-6–driven pathways most important in T1D pathogenesis.

## Methods

### Study design

This was a placebo-controlled, double-blinded, randomized clinical trial of individuals within 100 days of T1D diagnosis. Randomization was done through a central automated system. Both participants and study personnel were blinded to study treatment. Entry criteria included the presence of at least 1 diabetes-related autoantibody, peak stimulated C-peptide ≥ 0.2 pmol/mL during an MMTT, and absence of infections, malignancies, or hematologic abnormalities that could increase risk with tocilizumab administration. The study was powered to determine the effect of tocilizumab in pediatric participants. Adults (ages 18–45 years) were enrolled beginning in March 2015 to provide safety data prior to enrollment of children and to evaluate the impact of tocilizumab in this age group. Data on 35 adults who completed the 12-week postrandomization visit were reviewed by the DSMB and FDA in November 2016 and January 2017, respectively, prior to enrolling pediatric participants. The study was then opened to pediatric enrollment (ages 6–17 years), with the first pediatric participant enrolled on May 12, 2017. All participants were randomized within each age cohort to tocilizumab and placebo 2:1. The primary endpoint was the 2-hour C-peptide mAUC at week 52, adjusted for baseline. Participants were followed for a total of 2 years to evaluate safety and changes in immune response, but this manuscript summarizes the first 52 weeks of the trial for all participants. The full trial protocol for the EXTEND study is accessible through https://www.itntrialshare.org, a public website managed by the Immune Tolerance Network (ITN), with the creation of an account.

### Procedures

#### Study drug availability and administration.

For those with at least 30 kg body weight, tocilizumab or placebo was administered intravenously (IV) at a dose of 8 mg/kg to a maximum of 800 mg. For those weighing less than 30 kg, the dose was 10 mg/kg. Drug was administered every 4 weeks for 24 weeks for a total of 7 doses. For US sites, tocilizumab was donated by the manufacturer (Genentech, a subsidiary of Roche). For Australian sites, tocilizumab was purchased directly by the study site for use in the trial. Saline for infusion was used as placebo in both US and Australian sites.

#### Diabetes-related autoantibodies.

GAD, IA2, mIAA, and Znt8 antibodies were measured at screening by the Barbara Davis Center.

#### MMTT.

MMTTs were performed as previously described at screening and weeks 12, 24, 39, 52, 78, and 104 (41). In brief, individuals underwent testing before 10 am and after overnight fasting. Samples were obtained for glucose and C-peptide at 2 baseline time points at minutes –10 and 0, then at minutes 15 and 30, and then every 30 minutes for 2 hours. Those at least age 12 underwent a 4-hour MMTT at screening and weeks 52 and 104. C-peptide and glucose assays were run at the Northwest Lipid Research Laboratory (Seattle, Washington, USA) and University of Florida Health Pathology Laboratory (Gainesville, Florida, USA).

#### FSIVGTT.

FSIVGTT was conducted to measure insulin sensitivity in individuals at least age 15 years who agreed to this optional procedure at baseline and weeks 24 and 52 using methods as previously described (42). In brief, after fasting, 2 IV lines were placed, and baseline samples were drawn. A bolus of dextrose (11.4 g/body surface area m^2^) was given IV at time 0 over 2 to 3 minutes. Samples were drawn at minutes 2, 3, 4, 5, 6, 8, 10, 12, 14, 16, and 19. At 20 minutes, insulin (0.02 units/kg) was administered over 5 minutes. Samples continued to be collected from 22 to 180 minutes. The insulin sensitivity index was determined using Bergman’s minimal model (MINMOD Millennium; ref. 43).

#### Diabetes management.

All participants received intensive diabetes management. HbA1c was assessed at every study visit at a central lab, with the goal of treatment to meet age-specific American Diabetes Association targets without significant or severe hypoglycemia (44). Insulin usage data (total daily dose) was collected for the preceding 5 days before study visits.

#### CGM.

The use of CGM was optional for participants, and participants could use the study-provided Dexcom G4 sensor or a sensor of their choice used for their clinical care. CGM data were requested for 14 days prior to study visits at 0, 12, 24, 52, 78, and 104 weeks.

### Mechanistic assays

Mechanistic analysis was limited to pediatric participants who completed the primary outcome visit and who had a usable sample (see Supplemental Table 3 for numbers of patients analyzed for each assay at different time points).

#### Immunophenotyping, intracellular cytokine staining, and phosphoflow staining.

Cryopreserved PBMCs were thawed and stained with the ITN X-trial T cell phenotypic flow cytometry panel routinely used in ITN studies (6, 8, 45–47), a panel for IL-6R staining across cell types, an intracellular cytokine (ICS) panel, and a phosphoflow panel (Supplemental Table 4). FcX Block (BioLegend 4223301) treatment was performed prior to addition of all staining cocktails. Surface markers were stained using cocktails prior to eBioscience FOXP3 fix/perm (Thermo Fisher Scientific) for intracellular staining. Live/Dead Fixable Blue Dead Cell Stain (Thermo Fisher Scientific L23105) was performed prior to BD Phosflow Fix Buffer I (BD Biosciences 557870) and BD Phosflow Perm buffer III (BD Biosciences 588050) for phosphoflow. Two-hour stimulation with PMA and ionomycin for the ICS panel was performed in the presence of brefeldin A and monensin (BioLegend). Ten-minute stimulation with media alone or IL-6 (20 ng/mL) in X-VIVO 15 media (Lonza) was performed for phosphoflow, as performed previously (26).

All panels were acquired on an LSRFortessa with FACSDiva software (BD Biosciences) and analyzed with FlowJo software version 9.5 (Tree Star). To permit direct comparisons between samples acquired across days, instrument standardization was performed using 8 peak rainbow calibration beads (Spherotech), adjusting photomultiplier tube voltages so that seventh peak mean fluorescence intensities for each parameter were consistent. All samples from the same participant were run on the same day, and an internal control sample from 1 individual was run each week to identify any machine or staining issues. Gated populations with fewer than 100 events in immunophenotyping and fewer than 150 events in phosphoflow were excluded from analysis.

#### Treg suppression assay.

Samples from 10 randomly selected placebo-treated and 10 tocilizumab-treated pediatric participants with the greatest reduction in phosphorylated STAT3 with tocilizumab therapy were used in a Treg suppression assay. Teff cell resistance was determined by an in vitro Treg-mediated suppression assay using Teff cell surface expression of both CD25 and CD134 as a surrogate marker of Treg-mediated suppression (48). In brief, CD4^+^ T cells depleted of CD25^hi^ cells were isolated from PBMCs of 10 treated and 8 placebo pediatric patients, at baseline and weeks 12, 24, and 52, using a no-touch Miltenyi Biotec CD4 T Cell Isolation Kit II and positive Miltenyi Biotec CD25 Microbeads II prior to staining with CFSE (MilliporeSigma). CD4^+^CD25^+^CD127^lo^ Tregs from a single healthy donor were sorted, expanded, and frozen as described (48) and used as a constant source of Tregs for all suppression assays. CD4^+^CD25^dim^ T (Teff) cells were cultured at 100,000 cells per well. Tregs were added at ratios of 1:4, 1:8, 1:16, and 1:32 (Treg/Teff) and Dynabeads CD3/CD28 T Cell Expander bead (Life Technologies, Thermo Fisher Scientific) added at a ratio of 1:28 (beads/Teff cells). On day 2, Teff cells were stained (Supplemental Table 5). For analysis, Teff cells cultured in media alone were used to set gates for the various activation markers or proliferation. EF670 was used to identify Tregs. Percentage suppression (*s*) was calculated as follows: *s* = ([*a* − *b*]/*a)* × 100, where *a* is the percentage of CD25^+^CD134^+^ Teff cells in the absence of Tregs and *b* is the percentage of CD25^+^CD134^+^ Teff cells in the presence of Tregs. Samples were collected on a BD Biosciences FACSCanto II; data were analyzed using FlowJo V10.6.2 and GraphPad Prism.

#### Serum analysis.

Cytokine expression and sIL-6R were measured in serum samples at baseline and weeks 4, 12, 24, and 52 using the Mesoscale platform. In detail, IL-6, IFN-α, TARC, MDC, MIP1α, MIP1β, IL-1b, IL-7, IL-10, IL-12, IL-15, IL-17A, IL-8, IFN-γ, TNF-α, IFN-β, and IL-27 were measured through U-PLEX Human Biomarker Group 1 multiplex assay, and IL-6R was measured using the R-PLEX Human IL-6-R assay (Mesoscale).

#### Genotyping.

Genomic DNA from study participants was genotyped using the Axiom Precision Medicine Research Array (Thermo Fisher Scientific), consisting of 903,000 genome-wide and clinically relevant markers and more than 9000 markers across the HLA region for HLA imputation purposes. SNP genotypes were subjected to quality control using the Axiom Analysis Suite 3.0, and 856,419 markers passing quality metrics and Hardy-Weinberg equilibrium were selected for downstream analysis. All EXTEND samples were concordant for sex and yielded quality genome-wide SNP genotypes. The *IL6R* SNP rs2228145 was not included on the Axiom Precision Medicine array, and genotype was imputed from 2 SNPs, rs4129267 and rs61812598, that were in high linkage disequilibrium with rs2228145 (D′ 1.0, *r*^2^ 0.93–1.0) in all ancestries represented in the EXTEND participants. Genotypes at both SNPs were concordant and passed Hardy-Weinberg equilibrium.

### Statistics

#### Analysis of clinical data.

The primary endpoint, 2-hour C-peptide mAUC, was calculated using the trapezoidal rule and dividing by the duration of the MMTT (120 minutes). For this computation, the “time 0” C-peptide value was the average of C-peptide values measured at minutes –10 and 0. For “after time 0” time points, actual time points were used (e.g., 14 minutes instead of the prescribed 15 minutes) in the calculation of mAUC. If a C-peptide measurement was below the lower limit of detection (LLD), ½ the LLD was used.

The primary analysis of the primary endpoint used an ANCOVA model with change from screening to week 52 of the 2-hour C-peptide mAUC as the response and covariates of treatment, screening 2-hour C-peptide mAUC, and age. The primary analysis was done in the mITT sample on pediatric participants only. The mITT sample included randomized participants who received any study drug. For any participant who missed the week 52 MMTT assessment and whose last MMTT had at least 1 C-peptide result above the LLD, mAUC values were imputed using the estimates from a linear regression model with response of week 52 mAUC and covariates of age and mAUC at the most recent visit where mAUC was observed among participants in the same treatment arm. If all C-peptide time points from the most recent observed MMTT were below the LLD, then the last calculated mAUC was carried forward.

Secondary analyses for HbA1c and average insulin use per kilogram included ANCOVA models analogous to the primary analysis with no imputation for missing data. Furthermore, analogous ANCOVAs for C-peptide mAUC, HbA1c, and average insulin use per kilogram in adults and in the pooled cohort (pediatric and adult participants combined) were performed. In each age cohort and for each of C-peptide mAUC, HbA1c, and average insulin use per kilogram, mixed models using data from screening through week 52 were created. Covariates were treatment, study week ([MMTT assessment date – treatment start date]/7), age, treatment × study week, and age × study week. Random within-participant effects for intercept and slopes over time were included, and an unstructured covariance matrix was used. Fisher’s exact tests were used to compare the proportion of participants with a major hypoglycemic event through week 52, an infusion reaction, and hypersensitivity in each treatment group and age cohort.

For CGM data, summary statistics of mean, SD, coefficient of variation, and proportion of time in key ranges (<54, 54 to <70, <70, 70–180, >180 mg/dL) were calculated for each participant and visit. Sensor glucose values used in analysis came from the 2 weeks prior to a visit where at least 70% of the expected time points were available.

Minimal model analysis was used to calculate insulin sensitivity from the FSIVGTT results. For secondary and sensitivity efficacy analyses, corrections were not made for multiple comparisons. SAS version 9.4 was used for all analyses except for the FSIVGTT minimal model.

#### Sample size.

A target sample size of 78 pediatric participants was selected to detect a 39% improvement at week 52 in 2-hour C-peptide mAUC for tocilizumab over placebo using a 2:1 randomization with 80% power and a 2-sided test of significance at α = 0.05. This assumed a baseline C-peptide mAUC of 0.70 pmol/mL, root mean squared error (RMSE) of 0.22, a change of –0.31 pmol/mL in the placebo group, and a change of –0.158 pmol/mL in the treated group. Estimates of baseline mAUC, RMSE, and placebo group change came from 104 control group pediatric participants pooled from 5 new-onset T1D studies (4, 5, 31, 49, 50).

#### Analysis of mechanistic data.

Treatment group comparisons for the DC subpopulations, %suppression CD25^+^ of total CD4 at different Treg/Teff ratios, and serum IL-6 cytokines at a given visit, were performed using repeated measures 2-way ANOVA on log values, with baseline log values, visit, treatment, and visit × treatment as covariates. Treatment differences in the levels of IL-6R of total CD4, STAT3 phosphorylation in T cell subsets, and frequencies of Treg and Teff cell populations at each visit were analyzed with repeated measures 2-way ANOVA models on log values of fold change from baseline, controlling for visit, treatment, and visit × treatment. Tukey-Kramer post hoc tests were done for multiple-comparison adjustments. *P* values less than or equal to 0.05 were considered significant.

### Study approval

All studies were performed after local IRB approval at each institution. Participants provided written informed consent prior to the conduct of any study activities. In the case of minors, both parental consent and participant assent were obtained. Most study patients were enrolled in the United States; 5 pediatric participants were enrolled in Australia.

## Author contributions

CJG and JHB conceived the study; CJG, SL, SEG, DMW, JLG, KJG, WER, PR, AM, SMW, ET, LAD, KCH, WVM, RG, MH, MEC, DAS, DAB, HR, KMU, CLS, and SS collected the clinical data; KC, AL, HJN, and RT conducted the experiments; and CJG, JHB, KL, SAL, RT, ES, LJW, SK, KDB, HJN, LKE, KDB, JGM, and SS analyzed the data. CJG, JHB, RT, ES, SAL, LJW, and SS wrote the manuscript.

## Supplementary Material

Supplemental data

Trial reporting checklists

ICMJE disclosure forms

## Figures and Tables

**Table 1:**
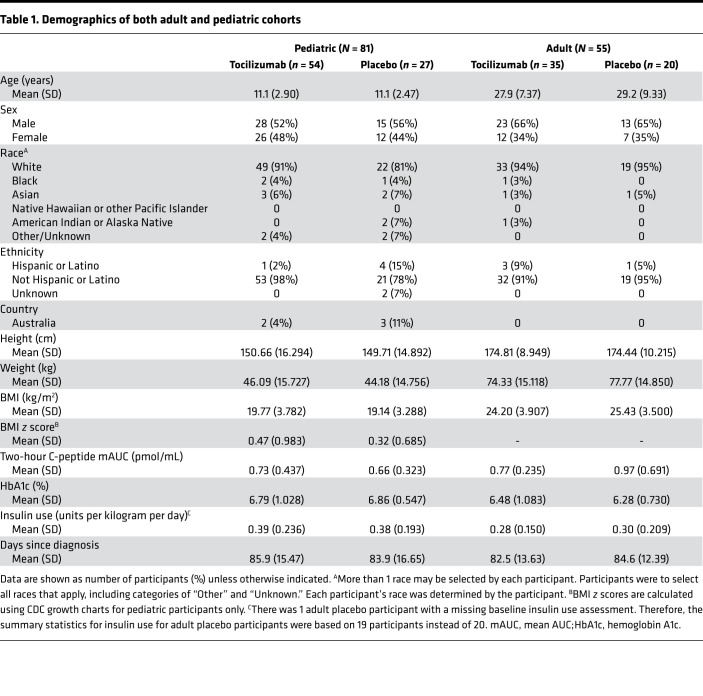
Demographics of both adult and pediatric cohorts

**Table 2:**
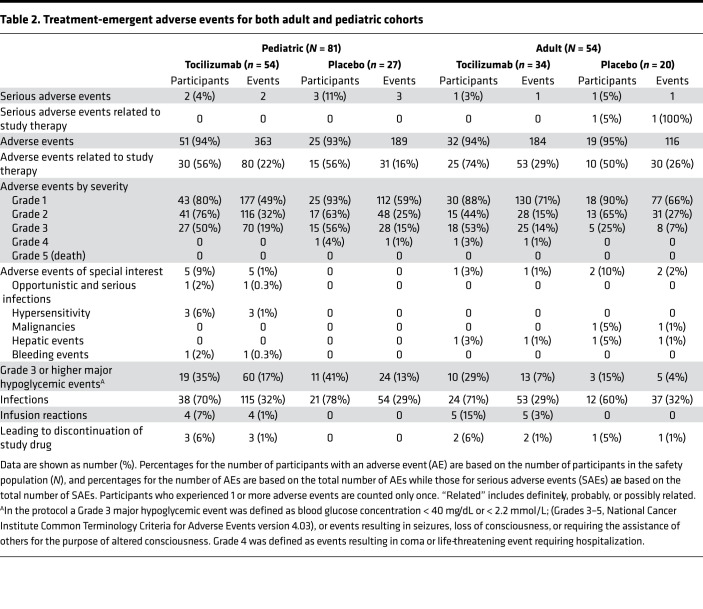
Treatment-emergent adverse events for both adult and pediatric cohorts

## Version 1 11/08/2021

Electronic publication
