# Supplementary material for: IL-6 receptor blockade does not slow β cell loss in new-onset type 1 diabetes
Source: JCI Insight. 2021 Nov 8;6(21):e150074. doi: 10.1172/jci.insight.150074 (PMC8663550; doi:10.1172/jci.insight.150074)

EXTEND Study Team Members:

ITN San Francisco: Mario Ehlers, Luke Lee, Preeti Chugha, Lauren McCarthy, Karen Smith, Audrey Plough, Srinath Sanda, Carol Soppe

ITN Bethesda: Michael Konieczny, Nabil Morad, Ligin Xu, Kristina Harris, Sai Kanaparthi, Elisavet Serti

ITN Seattle: Mary Roy, Alison Bemis, Sebastian Ramos, Shannon Achilles

NIAID: Peggy Lund-Fitzgibbon, Wendy Gao, Kasia Bourcier, Christine Czarniecki, Negin Atri, Sanaz Hamrah, Linna Ding, Peter Bianchine, Lyudmila Lyakh, James McNamara

Rho: Lynette Keyes-Elstein, Lasonia Morgan, Caroline Christman, Miguel Villarreal, Reggie Williams Kelly Lane, Susan McCahren Victoria Williams, Liz Goodman, Tom Kerby, Lia Winer, Karen Boyle

PPD: Brian Long, Charity-Anne Schullet, Sue Reddel, Murtaza Shipchandlier, Diana Guerrero, Jessica Steward, Mary Glessner, Heather Daview, Reena Pandya, Penelope Kopf, Katie Tayloe, Jeffery Smith, Lindsay Tollefson, Cheryl Wilmonth, Veronica Grimes, Judy Shepard, Tonya Ezzelle

Benaroya Research Institute: Marli McCulloch-Olson, Jenna Snavely, Mary Ramey, Megan Tobin, Thien-Son Ngyuen, Pamela Miranda, Cameron Willman, Anna Barash, Craig Pedersen, Bobby Noble, Amy Lee, Wendy Williker, Linda Dement, Hannah Klehr, Elaine Sachter, Sheila Scheiding, Cherry Fletcher, Dylan Gnatz, Kalyn Lehner, Nan Roa, Manj Randhawa, Deborah Hefty, Rachel Hartley, Lisa Miller, Christine Webber, Sarah Robinson, Corinna Tordillos, Alice Long, Kathi Lambert, Karen Cerosaletti, Jane Buckner, Carla Greenbaum, Sandra Lord

Columbia University: Eleanor Zagorean, Rocio Pena, Marta Scotto, Lucy Liu, Connie Eng, Donalrey Nieva, Donalrey Nieva, Elisabeth Robinson, Analia Alvarez, Jacqueline Jezioro, Janelle Rivera, Samantha Azinge, Magdalena Bogun, Kristen Williams, Sarah Pollak, Robin Goland

Indiana University: Brittany Weaver, Melissa Allard, Denise Cox, Jana Effinger, Molly Duncan, Maureen Mullen, Alicia Hedges, Jessica Anders, Robin Hufferd, David Hansen, Devyn Rae Purtlebaugh, Brianne Kost, Erin Dykstra, America Newnum, Juan Sanchez, Carmella Molina, Emily Sims, Stephanie Woerner, Megan Hildinger, Ellie Ryan, Eric Grubbs, Heba Ismail, Maria Nicholson Spall, Linda DiMeglio

Joslin: Gordon Weir, Jeanne Turley, Laurie Higgins, Joshua Kasper, Pamela Ariniello, Alissa Segal, Michelle Taiby, Nisha Koshy, Suzanne Krishfield, Nora Bryant, Brittany Resnick, Jason Gaglia

Sanford Research: John Shelso, Alaa Al Nofal, Karen Hanishch, Ann Mays, Dawn Hanson, Hanna Williams, Branden Pfaff, Lacey DeGeest, Kristi Atkins, Troy Hollinsworth, Brooke Kappenman, Ashley Hopp, Collette Springman, Tyler Turek, Thaddaus Hellwig, Kurt Griffin

Stanford University: Tandy Aye, Hilary Seeley, Priya Prahalad, Ananta Addala, Laila Craveiro, Karen Brahona, Bonnie Baker, Trudy Esrey, Darrell Wilson

UCSF: Sara Stillwell, Shirley Chen, Christine Ferrara, Beverly Non-Garcia, Diana Ng Wong, Wendy Staub, Christine Torok, Sheila Ghods, Ella Ly, Ashley Cook, Ronald Honrada, Nicholas

Larocque, Tracy Rodriguez, Glenna Auerback, Bruce Lilley, Maharlika Abalos, Zulma Finney, Airica Bustos, Rebecca Wesch, Steve Gitelman

University of Minnesota: Adi Molvin, Beth Pappenfus, Lori Wallin, Darlette Luke, Derek LaBar, Theresa Christiansen, Alyssa Halper, Brandon Nathan, Melena Bellin, Janice Leschyshyn, Jessica Sweet Ruedy, Anne Street, Nancy Vang, Brittney Nelson, Avery Bartyzal, Darcy Weingartner, Jennifer McVean, Toni Moran

University of Florida: Jennifer Hosford, Tomy Matthew, Janet King, Norma Kerr, Glenda Ellis, Susan Beltz, Melissa Johnson, Elizabeth Agustin, Laura Jacobsen, Kenneth Cusi, Janet Silverstein, Annie Araham, Michele Romano, Jamie Thomas, Miriam Cintron, Michael Haller, Henry Rohrs, Des Schatz

University of Texas Southwestern: Lauren Ziemian Boyles, Maria Lourdes Pruneda, Dana Hightower, Laura Breen, Michael Phan, Tram Meeks, Brian Kreymer, Serey Sao, Philip Raskin

University of Miami: Della Matheson, Carlos Blaschke, Burlett Masters, Juan Perez-Scholz, Anjanette Arzani, Eric Zetka, Jeremy Deni, Daniel Nobel, Ana Romero, Irlisse Couvertier, Jennifer Marks, Jay Skyler, Janine Sanchez, David Baidal

University of South Florida: Emily Eyth, Oliver Emberger, Mark Pennington, Danielle Henson, Dorthy Shulman, Sureka Bollepalli, Michele Laine, Henry Rodriguez

Vanderbilt University: Anne Brown Faith Brendle, George Williams, John Oleis, Dan Moore, Justin Gegory, Bill Russell

Yale University: Linda Ryall, Angeliki Maria Stamatouli, Jean Mack-Fogg, Laurie Feldman, Naomi Yama, Assad Awan Osama Abdelghany, Debra Moss, Eileen Mydosh, Leslie Laich, Tenzin Kunzom, Cathy Canny, Ana Perdigoto, Laura Cataldi-Young, Linda Rink, Kevan Herold

Children's Hospital of Philadelphia: Fiona Stuart, Oksana Khavunka, Diana Olivos, Jennifer Smith, Audrey Kamrin, Catherine Carchidi, Brian Grant, Olena Kucheruk, Sooji Lee, Patrick Hanley, Carmen Carrasquillo, Brigid Czyszczon, Steven Willi

University of Iowa: Joanne Cabbage, Michael Tansey, Julie Coffey, Rachel Bisbee, Jessica Gallagher, Jackline Wanui-Very, Kristine Johnson, Theresa Hobbs, Wendi Slaughter, Jacob Hampton, Amanda Schaapveld, Molly Martin, Stacy McMichael, Stephanie Rosazza, Eva Tsalikian

Children's Mercy Hospital: Marissa Beidelschies, Jennifer Dolan, Emily Haith, Anne Clark, Dara Watkins, Kori Hess, Jennifer Boyd, Ryan McDonough, Terri Luetjen, Jennifer Boyd, Kelsee Halpin, Wayne Moore

Children's Hospital Westmead: Andrew Biggins, Amy Bruce, Lily Nahidi, Natalie Stefanic, Jacki Catteau, Emily Wood, Jessica Bolitho, Emily Gibson, Amy Boland, Violet Ford, Youstiena Soliman, Amina Shah, Shylaha Immadi, Susanne Chen, Sophie Brown, Marcus Vallejo, Nacha Han, Kim Donaghue, Eschwini Tadiyal, Anthea Ng, Carolyn Casey, Maria Craig

University of Queensland: Tracey Baskerville, Myra Toonen, Matthew Spierings, Wendy Hamilton, Peng Choy, Lisa Bidwell, Anita Chamion, Rochelle Green, Julie Kirby, Jerry Wales, Samuel Blake, Evonne Smith, Yasmin Musthaffa, Andrey Cotterill, Mark Harris

Supplemental Table 1: Continuous glucose monitoring data. CGM data for the pediatric cohort is shown with summary statistics including mean glucose, SD, CV, and percent of reading in various glucose ranges shown.

	Pediatric (N=81)	
	Tocilizumab (N=54)	Placebo (N=27)
Baseline		
n	6	7
Mean: Mean (SD)	165.7 (35.19)	159.5 (23.73)
SD: Mean (SD)	54.4 (14.34)	54.3 (16.86)
CV: Mean (SD)	32.7 (5.32)	33.4 (6.91)
% Readings in Range >180 mg/dL: Mean (SD)	33.4 (22.73)	28.1 (16.11)
% Readings in Range 70-180 mg/dL: Mean (SD)	65.5 (22.00)	71.3 (16.24)
% Readings in Range <70 mg/dL: Mean (SD)	1.1 (0.80)	0.6 (0.51)
% Readings in Range 54-<70 mg/dL: Mean (SD)	0.9 (0.72)	0.5 (0.46)
% Readings in Range <54 mg/dL: Mean (SD)	0.1 (0.12)	0.1 (0.09)
Week 4		
n	15	6
Mean: Mean (SD)	148.0 (32.44)	170.2 (22.14)
SD: Mean (SD)	46.6 (15.90)	61.0 (7.46)
CV: Mean (SD)	30.9 (6.12)	36.2 (4.78)
% Readings in Range >180 mg/dL: Mean (SD)	22.6 (19.18)	38.6 (14.06)
% Readings in Range 70-180 mg/dL: Mean (SD)	76.1 (18.49)	59.7 (12.01)
% Readings in Range <70 mg/dL: Mean (SD)	1.2 (1.33)	1.7 (2.62)
% Readings in Range 54-<70 mg/dL: Mean (SD)	1.1 (1.19)	1.5 (2.17)
% Readings in Range <54 mg/dL: Mean (SD)	0.1 (0.17)	0.2 (0.46)
Week 8		
n	12	7
Mean: Mean (SD)	156.4 (29.69)	180.0 (31.51)
SD: Mean (SD)	49.7 (15.07)	62.7 (16.48)
CV: Mean (SD)	31.6 (7.04)	34.5 (6.62)
% Readings in Range >180 mg/dL: Mean (SD)	28.7 (18.14)	43.3 (19.77)
% Readings in Range 70-180 mg/dL: Mean (SD)	70.1 (17.87)	55.6 (19.57)
% Readings in Range <70 mg/dL: Mean (SD)	1.2 (1.70)	1.1 (1.26)
% Readings in Range 54-<70 mg/dL: Mean (SD)	1.0 (1.21)	0.9 (0.93)
% Readings in Range <54 mg/dL: Mean (SD)	0.2 (0.59)	0.2 (0.34)
Week 12		
n	17	8
Mean: Mean (SD)	156.3 (27.22)	180.0 (29.78)
SD: Mean (SD)	49.2 (11.58)	65.1 (18.01)

CV: Mean (SD)	31.4 (5.45)	35.8 (5.88)
% Readings in Range >180 mg/dL: Mean (SD)	29.2 (19.29)	42.9 (17.59)
% Readings in Range 70-180 mg/dL: Mean (SD)	69.8 (18.97)	55.6 (17.59)
% Readings in Range <70 mg/dL: Mean (SD)	0.9 (0.90)	1.5 (1.53)
% Readings in Range 54-<70 mg/dL: Mean (SD)	0.8 (0.75)	1.2 (1.23)
% Readings in Range <54 mg/dL: Mean (SD)	0.1 (0.23)	0.3 (0.33)
Week 16		
n	24	9
Mean: Mean (SD)	157.7 (30.35)	163.4 (38.64)
SD: Mean (SD)	51.8 (17.35)	57.8 (17.10)
CV: Mean (SD)	32.3 (5.68)	35.3 (7.27)
% Readings in Range >180 mg/dL: Mean (SD)	28.8 (18.69)	33.8 (22.81)
% Readings in Range 70-180 mg/dL: Mean (SD)	69.6 (18.54)	64.3 (21.93)
% Readings in Range <70 mg/dL: Mean (SD)	1.6 (1.93)	1.9 (1.67)
% Readings in Range 54-<70 mg/dL: Mean (SD)	1.2 (1.10)	1.6 (1.41)
% Readings in Range <54 mg/dL: Mean (SD)	0.4 (0.90)	0.3 (0.29)
Week 20		
n	25	7
Mean: Mean (SD)	159.2 (29.84)	156.5 (21.46)
SD: Mean (SD)	51.4 (14.60)	58.0 (12.94)
CV: Mean (SD)	31.9 (5.05)	37.0 (5.76)
% Readings in Range >180 mg/dL: Mean (SD)	29.3 (18.40)	31.0 (12.72)
% Readings in Range 70-180 mg/dL: Mean (SD)	69.7 (18.29)	66.5 (12.58)
% Readings in Range <70 mg/dL: Mean (SD)	1.0 (1.28)	2.5 (2.22)
% Readings in Range 54-<70 mg/dL: Mean (SD)	0.8 (0.85)	1.9 (1.73)
% Readings in Range <54 mg/dL: Mean (SD)	0.2 (0.48)	0.5 (0.68)
Week 24		
n	22	11
Mean: Mean (SD)	159.9 (25.89)	167.4 (28.35)
SD: Mean (SD)	53.1 (15.13)	62.3 (15.39)
CV: Mean (SD)	32.8 (5.12)	36.9 (4.84)
% Readings in Range >180 mg/dL: Mean (SD)	29.8 (15.20)	35.1 (17.44)
% Readings in Range 70-180 mg/dL: Mean (SD)	69.3 (15.00)	63.6 (17.41)
% Readings in Range <70 mg/dL: Mean (SD)	0.9 (0.90)	1.3 (0.86)
% Readings in Range 54-<70 mg/dL: Mean (SD)	0.8 (0.82)	1.1 (0.68)
% Readings in Range <54 mg/dL: Mean (SD)	0.1 (0.13)	0.2 (0.22)
Week 39		
n	28	10
Mean: Mean (SD)	164.7 (22.22)	183.7 (50.90)
SD: Mean (SD)	58.6 (13.93)	65.9 (18.39)
CV: Mean (SD)	35.5 (6.36)	36.3 (6.72)
% Readings in Range >180 mg/dL: Mean (SD)	34.7 (14.51)	42.1 (24.30)

% Readings in Range 70-180 mg/dL: Mean (SD)	63.1 (14.26)	56.2 (23.51)
% Readings in Range <70 mg/dL: Mean (SD)	2.2 (2.98)	1.7 (2.04)
% Readings in Range 54-<70 mg/dL: Mean (SD)	1.8 (2.37)	1.4 (1.57)
% Readings in Range <54 mg/dL: Mean (SD)	0.4 (0.67)	0.3 (0.51)
Week 52		
n	32	9
Mean: Mean (SD)	167.1 (28.11)	182.0 (46.43)
SD: Mean (SD)	58.4 (15.00)	67.6 (16.61)
CV: Mean (SD)	34.8 (4.96)	37.6 (6.97)
% Readings in Range >180 mg/dL: Mean (SD)	35.4 (16.40)	42.3 (20.11)
% Readings in Range 70-180 mg/dL: Mean (SD)	63.0 (16.01)	55.2 (19.37)
% Readings in Range <70 mg/dL: Mean (SD)	1.6 (1.66)	2.5 (2.66)
% Readings in Range 54-<70 mg/dL: Mean (SD)	1.3 (1.07)	2.1 (2.05)
% Readings in Range <54 mg/dL: Mean (SD)	0.3 (0.69)	0.4 (0.64)

Supplemental Table 2: Genotype frequencies in both adult and pediatric cohorts.

Pediatric Patients				
Treatment Group	(rs61812598)			
	AA	AG	GG	Total
Placebo	5	10	9	24
Tocilizumab	5	27	20	52
Total	10	37	29	76
	13.16	48.68	38.16	100
Fisher's exact p-value: 0.405				

Pediatric Patients				
Treatment Group	(rs4129267)			
	TT	CT	cc	Total
Placebo	5	10	9	24
Tocilizumab	5	27	20	52
Total	10	37	29	76
	13.16	48.68	38.16	100
Fisher's exact p-value: 0.405				

Pediatric Patients				
Treatment Group	(rs4129267)			
	TT	CT	CC	Total
Placebo	5	10	9	24
Tocilizumab	5	27	20	52
Total	10	37	29	76
	13.16	48.68	38.16	100
Fisher's exact p-value: 0.405				

Adult Patients				
Treatment Group	(rs4129267)			
	TT	CT	CC	Total
Placebo	3	9	8	20
Tocilizumab	10	16	8	34
Total	13	25	16	54
	24.07	46.3	29.63	100
Fisher's exact p-value: 0.341				

Supplemental Table 3: Participants studied for each mechanistic assay

**Cell population**	**Treatment**	**Week 0**	**Week 4**	**Week 12**	**Week 24**	**Week 52**
MFI pSTAT3 of CD4 Teff	Placebo	22	19	22	22	22
MFI pSTAT3 of CD4 Teff	Tocilizumab	43	41	43	43	42
% total pSTAT3 of Tregs	Placebo	22	18	21	21	22
% total pSTAT3 of Tregs	Tocilizumab	42	40	40	42	41
%pSTAT3 of CD4 Memory Teff	Placebo	21	18	21	21	21
%pSTAT3 of CD4 Memory Teff	Tocilizumab	43	41	43	43	42
MFI IL6 of Total CD4	Placebo	22	19	22	22	22
MFI IL6 of Total CD4	Tocilizumab	43	41	43	43	42
% CD45RO+ (memory) of CD4 Teff	Placebo	25	23	25	25	24
% CD45RO+ (memory) of CD4 Teff	Tocilizumab	49	47	49	49	48
%CCR7+ (CM) of memory CD4 Teff	Placebo	25	23	25	25	24
%CCR7+ (CM) of memory CD4 Teff	Tocilizumab	49	47	49	49	48
%CCR7- (EM) of CD4 Teff	Placebo	25	23	25	25	24
%CCR7- (EM) of CD4 Teff	Tocilizumab	49	47	49	49	48
%Treg (FoxP3+) of CD4 T cells	Placebo	25	23	25	25	24
%Treg (FoxP3+) of CD4 T cells	Tocilizumab	49	47	49	49	48
% IL-21+ of FoxP3-CD45RO+	Placebo	26	24	26	26	25
% IL-21+ of FoxP3-CD45RO+	Tocilizumab	53	50	53	53	52
% IL-17a of FoxP3-CD45RO+	Placebo	26	24	26	26	25
% IL-17a of FoxP3-CD45RO+	Tocilizumab	53	50	53	53	52
cDC1	Placebo	25	25	25	23	24
cDC1	Tocilizumab	49	49	48	47	48
cDC2	Placebo	25	25	25	23	24
cDC2	Tocilizumab	49	49	48	47	48
pDC	Placebo	25	25	24	23	24
pDC	Tocilizumab	49	49	48	47	48
Classical Monocytes	Placebo	25	25	25	23	24
Classical Monocytes	Tocilizumab	49	49	48	47	48
**Treg Suppression Assay**	-	-	-	-	-	-
**Treatment**	**TReg:teff Ratio**	**Week 0**	**Week 4**	**Week 12**	**Week 24**	**Week 52**
Placebo	1:4	8	10	10	10	9
Tocilizumab	1:4	10	10	10	10	10
Placebo	1:8	10	10	10	10	10
Tocilizumab	1:8	10	10	10	10	10
Placebo	1:16	10	10	10	10	10
Tocilizumab	1:16	10	10	10	10	10
Placebo	1:32	9	10	10	10	9
Tocilizumab	1:32	10	10	10	10	10

**Serum Analyte**	**Treatment**	**Week 0**	**Week 4**	**Week 12**	**Week 24**	**Week 52S**
CRP	Placebo	25	25	25	25	25
CRP	Tocilizumab	49	49	49	49	49
IL6	Placebo	23	23		23	21
IL6	Tocilizumab	43	47		47	40
sIL6-R	Placebo	23	23		22	22
sIL6-R	Tocilizumab	47	47		47	46

Supplemental Table 4: Flow cytometry staining panels used for immunophenotyping

ITN X-trial T cell panel		
Marker	Fluorochrome	Clone
CD3	BV605	OKT3
CD4	BB515	RPA-T4
CD8	AlexaFluor700	SK1
CD56	BUV395	NCAM16.2
CCR7	BV510	G043H7
CD45RA	BUV737	Hl100
CD45RO	BV786	UCHL1
CD127	BV711	A019D5
FoxP3	PE-CF594	259D/C7
PD1	BV650	EH12.2H7
CD57	APC-Vio770	REA769
KLRG1	PE-Cy7	SA231A2
Eomes	PE	WD1928
TIGIT	APC	MBSA43
Ki67	BV421	Ki67
CD95	BB700	DX2
viability	BUV496	

ICS panel		
Marker	Fluorochrome	Clone
CD3	Alexa Fluor 700	OKT3
CD4	BV650	SK3
CD8	BUV737	SK1
CD45RA	BV605	HI100
CXCR5	BV785	J252D4
PD1	BV421	EH12.2H7
CD56	PE-CY7	NCAM16.2
CD161	BV510	HP-3G10
FoxP3	PE-CF594	259D/C7
CRTH2	BV711	BM16
CD45RO	BB515	UCHL1
IFNg	BUV395	B27
TIGIT	APC	MBSA43
IL21	PE	3A3-N2.1
IL17A	APC-Cy7	BL168
viability	BUV496	

IL6-R / APC panel		
Marker	Fluorochrome	Clone
CD19	BV421	HIB19
CD141	BB700	1A4
CD3	BUV737	UCHT1
HLADR	BV510	G46-6
CD123	PE	7G3
CD1c	BV650	F10/21A3
CD16	APC-H7	3G8
CD8	FITC	RPA-T8

CD4	AF700	RPA-T4
CD14	BV786	M5E2
IgD	BV605	IA6-2
CD56	PE-CF594	NCAM16.2
CD38	APC	HIT2
CD27	BV711	M-T271
IL6-R	PE-Cy7	UV4
viability	BUV496	

Phospho-flow Panel		
Marker	Fluorochrome	Clone
CD3	PerCP-Cy5.5	UCHT1
CD4	BV605	RPA-T4
CD8	BUV395	RPA-T8
CD45RA	BUV737	Hl100
CD27	BV510	O323
CD56	PE-CY7	NCAM16.2
CD33	APC	P67.6
CD25	PE-CF594	M-A251
CD127	APCef780 (H7)	RDR5
pSTAT3 (Y705)	PE	4/pSTAT3
pSTAT1 (Y701)	AF488	4a
CD45RO	BV786	UCHL1
viability	BUV496	

Supplemental Table 5: Flow cytometry staining panel used for Treg suppression assay

Marker	Fluorochrome	Catalog #
CD3	BV421	Biolegend 300434
CD4	APC-Cy7	BD 557871
CD45RO	BV605	BD562790
CD45RA	FITC	Biolegend 983002
CD25	PE-Cy7	BD557741
CD134	PE	BD555838

Supplemental Figure 1: Lipid levels. Markers represent the means, lines connect the medians, and error bars represent the 25th and 75th percentiles. Longitudinal lipid levels observed in both cohorts for total cholesterols (A and B), HDL (C and D), and LDL (E and F).

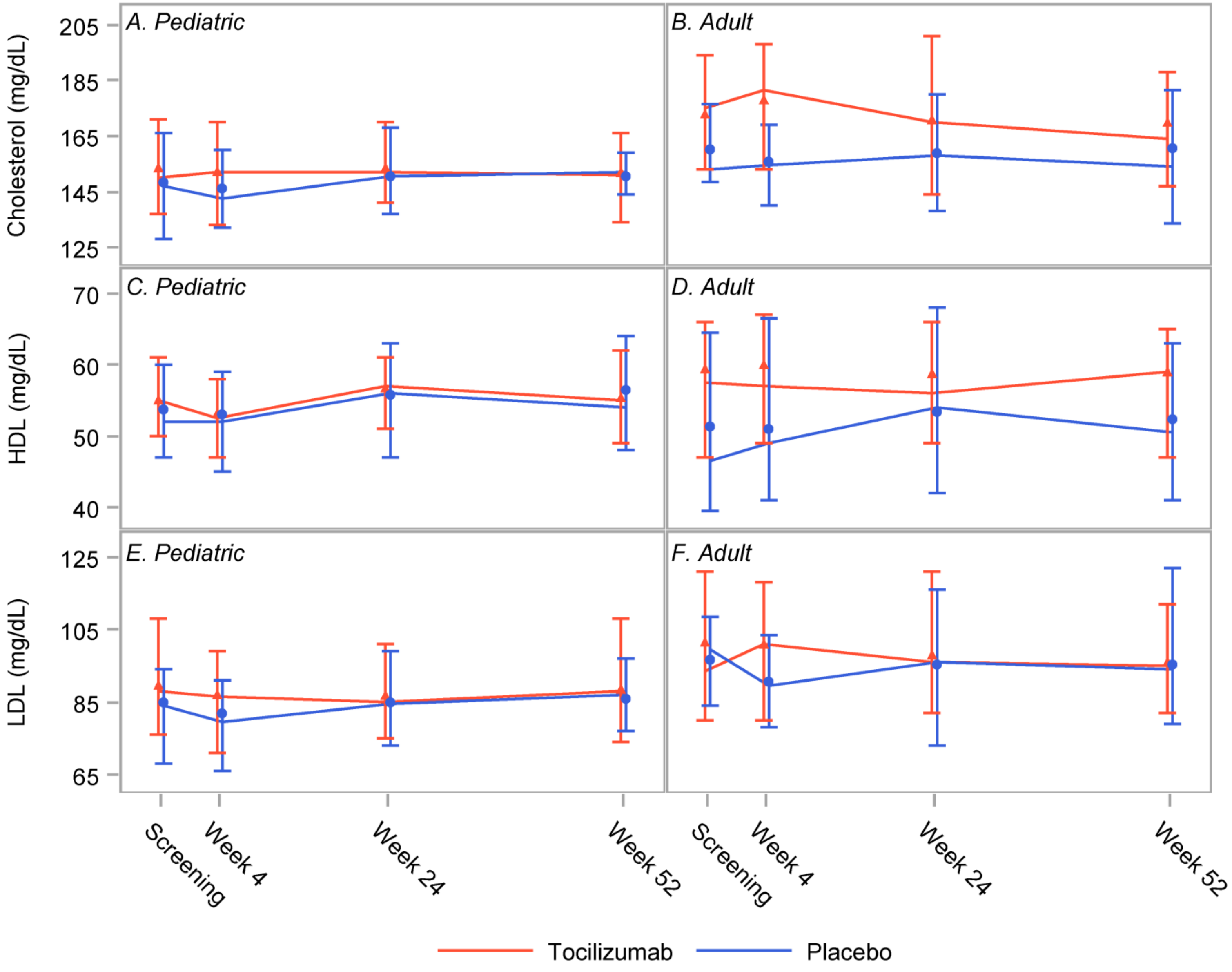

Supplemental Figure 2: Longitudinal changes in 4-hour C-peptide mAUC.

4-hour C-peptide mAUC. Markers represent the means, lines connect the medians, and error bars represent the 25th and 75th percentiles of C-peptide mAUC collected during the 4-hour MMTT for participants >=12 years of age. Data shown at screening and week 52 for (A) the pediatric cohort and (B) the adult cohort. Not all pediatric subjects received a 4-hour MMTT; in the pediatric cohort a total of 27 tocilizumab treated participants had a 4-hour MMTT at screening and 26 had a 4-hour MMTT at week 52. In the placebo group, 9 patients had a 4 hour MMTT at screening and 12 at week 52.

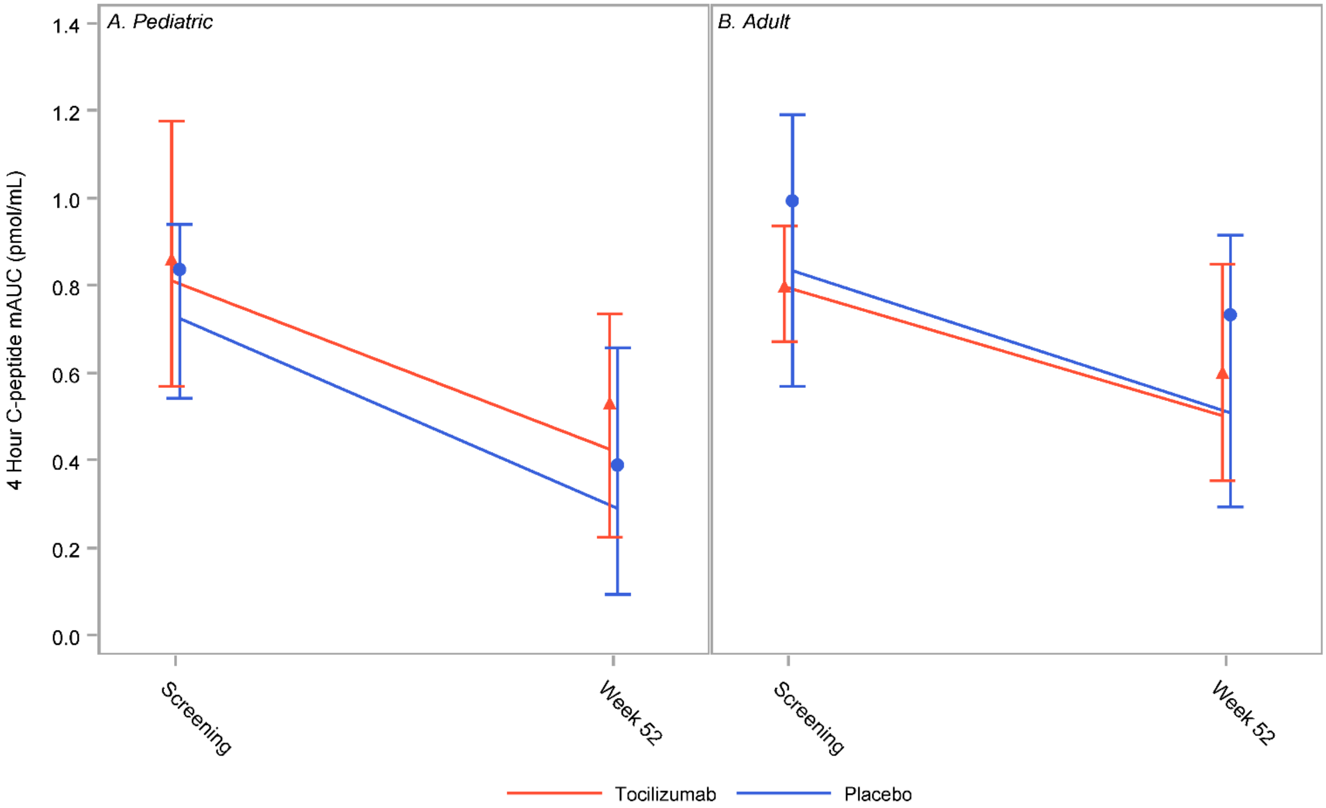

Supplemental Figure 3:

Insulin sensitivity (SI) from FSIVGTT. SI over time is displayed for each subject who participated in the FSIVGTT assessments.

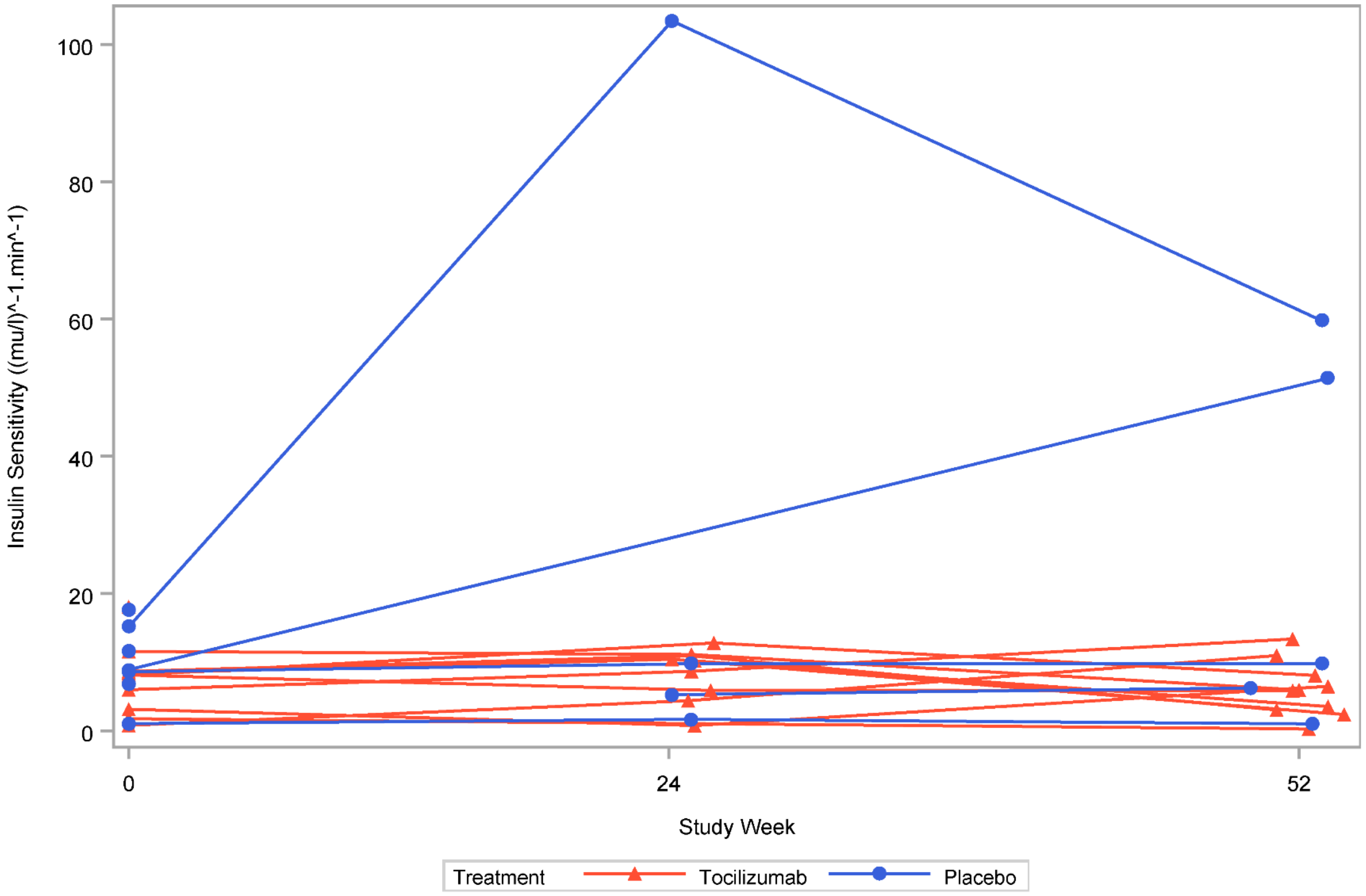

Supplement: Supplemental data [file jciinsight-6-150074-s063.pdf]
